# The ventrolateral prefrontal cortex is part of the modular working memory system: A functional neuroanatomical perspective

**DOI:** 10.3389/fnana.2023.1076095

**Published:** 2023-02-27

**Authors:** Orin Segal, Odelia Elkana

**Affiliations:** School of Behavioral Sciences, The Academic College of Tel Aviv Yaffo, Tel Aviv, Israel

**Keywords:** vlPFC, ventrolateral prefrontal cortex, working memory, functional neuroanatomical framework, lateral OFC, inattentiveness, PFC, selective attention

## Abstract

For many years, the functional role of the ventrolateral Pre-Frontal Cortex (PFC) was associated with executive functions, specifically in the context of non-affective cognitive processes. However, recent research has suggested that the ventrolateral PFC is also involved in the attention system. The Ben Shalom model of the functional organization of the prefrontal cortex (2019) posits that the ventrolateral PFC selects perceptual stimuli after integration by the adjacent ventromedial PFC. This article reviews the state-of-the-art findings to better understand the role of the ventrolateral PFC in the selection of perceptual information as grounded in the Ben Shalom model. Numerous studies have reported converging evidence for the selective role of this area. However, most argue that this perceptual selection takes place through the active updating of information values linked to goal-oriented actions. These studies thus view the ventrolateral PFC as part of a system that actively manipulates and changes processed information such as the working memory function, rather than being part of the attention system. In agreement with this view, this review suggests that this area is part of a complex and modular working memory system and illustrates with reference to Diamond’s work on ADD. This working memory system is functionally and anatomically dispersed and includes the dorsolateral PFC, the ACC, the parietal cortex, the basal ganglia, and the cerebellum. Hence, future research should continue to explore the specific neurofunctional roles of these areas in working memory systems, and the connections between the different subareas in this complex array.

## Introduction

The frontal lobes and specifically the Pre-Frontal Cortex (PFC) are considered to mediate executive functions; i.e., the range of mental functions guiding human behavior *via* the coordination, operation, and integration of more basic mental processes (Ward, 2020). Although there is a general consensus that the PFC plays a major role that underlies these executive functions, the ways in which they are related to the anatomical structure of the PFC is still hotly debated. Different models have been put forward to clarify this functional neuroanatomical association. Most theories derive from one broader model that makes a horizontal distinction between the lateral PFC and the medial PFC (Ward, 2020) which distinguishes between affective and nonaffective executive functions. The medial PFC, which includes the ventral orbitofrontal cortex, is thought to be involved in the processing of emotional and social stimuli, and reward-related stimuli in particular, whereas the lateral PFC is believed to be involved in pure emotionally-neutral cognitive, sensory-related stimuli processing (Ward, 2020).

However, other models have been proposed, suggesting different hierarchies of information processing along different axses, such as anterior-posterior or dorsal-ventral (Ben Shalom and Bonneh, 2019; e.g., Koechlin and Summerfield, 2007). Ben Shalom (2009) and Ben Shalom and Bonneh (2019) proposed a framework including both a horizontal and a vertical distinction. In this model, the PFC is functionally organized in four different subareas where Brodmann areas BA 11 and 47, corresponding to the Orbitofrontal Cortex (OFC) and the inferior frontal gyri, are involved in perception, BA 10 and 46, roughly corresponding to the middle frontal gyri, are involved in memory, BA 9, comprising the dorsal regions of the PFC, is involved in emotion and BA 8, in the superior frontal gyri and posterior to BA9, is involved in motor information (Ben Shalom, 2009; Ben Shalom and Bonneh, 2019). While the original model (Ben Shalom, 2009) focused on vertical neuro-functional organization, in 2018, Ronel suggested that while the subareas in both the medial and lateral PFC process similar types of information (sensation perception, memory, emotion, and motor), they also have specific functions, where the medial division integrates subcortical and cortical—sensory and cognitive information, the lateral counterpart is involved in the selection and inhibition of this information (Ronel, 2018).

Here, we extend the Ronel and Ben Shalom model (Ronel, 2018; Ben Shalom and Bonneh, 2019) to explore the role of BA 47 and the lateral BA 11. Ben Shalom’s model describes these anatomical subareas using the Brodmann classification. However, most current functional neuroanatomical literature on the PFC uses other taxonomies, such as cerebral divisions into sulci and gyri, or a simple division of the PFC into the four ventromedial, ventrolateral, dorsomedial, and dorsolateral areas. We use this definition when referring to BA 11 and 47. In what follows the more general term PFC or the specific term OFC is used when appropriate.

## The lateral OFC: selection and inhibition of perceptual information or goal-directed guidance?

Ronel presents experimental evidence for the role of the ventrolateral PFC, and especially the lateral OFC, in perceptual selection processes (Ronel, 2018). Ronel suggests that the lateral OFC is involved in assigning and updating selection criteria according to stimulus values, rejecting irrelevant stimuli, and maintaining relevant information in working memory.

There is evidence that the lateral OFC plays a role in task-specific and goal-directed information selection (Gremel and Costa, 2013; Zsuga et al., 2016; Ronel, 2018; Malvaez et al., 2019). However, it is difficult to clearly distinguish between selection functions and the other more integrative perceptual processes needed for the goal-directed guidance in which this subarea is involved. For example, recent studies have indicated that the lateral OFC is involved in the updating of outcome values and integrating specific external and internal perceptual presentations to achieve a goal (Baltz et al., 2018; Stayte et al., 2021). These studies lend weight to its putative perceptual role but do not differentiate the lateral and medial parts with respect to the integrative role that was suggested to be under the control of the medial areas in Ben Shalom’s model. Moreover, many studies on the functional properties of this area continue to stress its role in goal-directed behavior, including its involvement in goal-directed cognitive control processes, but put forward different mechanisms to underlie this function (e.g., Tang et al., 2016; Sadacca et al., 2018; Wallis, 2019; Tripathi et al., 2021). Although the literature tends to confirm Ronel and Ban Shalom’s claim that the ventral PFC, including both the medial and lateral OFC, is closely involved in the processing of perceptual information, the role of the lateral OFC in goal-directed behavior, and how the processing of perceptual information is related to this, remain unclear.

What further complicates the issue is that the OFC is hypothesized to be involved in acquiring information to infer the subjective and emotional value of actions (Rich and Wallis, 2016). That is, its selection properties are part of a learning process where action values are constantly updated by preferring or rejecting the perceptual stimulus related to the updated value outcomes. To do so, the OFC’s main function is thought to be driven by behavior-reward associative learning (Kennerley and Wallis, 2009; Zsuga et al., 2016; Sadacca et al., 2018; Knudsen and Wallis, 2020). Studies have reported the existence of neural connections between the OFC, the ventral striatum, and the thalamus in humans and primates, thus suggesting that the OFC plays a role in reward learning (Balleine and O’Doherty, 2010). It is further hypothesized that in this cortico-striatum learning loop, the ventral striatum enables a fast reward learning route while the cortex balances this route in a slower, more gradual learning route, integrating different past and present representations (Buschman et al., 2014). Other studies that have recorded neurons in the rat indicate neural activation in the OFC subsequent to reward training but also after non-rewarding stimulus associations (Sadacca et al., 2018). Following this line, some studies, suggest that while the OFC, in general, represents and updates the emotional value of information, there is a different function between the medial the lateral sub area’s functions. While the medial OFC represents expected rewards, the lateral OFC represents non-reward and punishment values (Rolls et al., 2020; Xie et al., 2021). It is important to note that while many studies agree that the lateral OFC has an important role in updating values, many believe it holds both reward and non-reward values (Sescousse et al., 2010; Malvaez et al., 2019).

While many studies have dealt with the role of OFC in the association between value and action during goal-directed behavior and consider that the lateral OFC is involved in the selection or rejection of sensory-perceptual information, they do not suggest that this is its main role. Instead, most have pointed to higher functions such as action selection, memory, and information integration. One possible explanation for the apparent discrepancy with Ben Shalom and Ronel’s perceptual processing hypothesis of the broader ventral PFC area is that the lateral OFC, which is a subarea of the ventrolateral PFC, may reciprocally select and process sensory-perceptual information, and update its value through Pavlovian and operant associations, thus actively seeking value-related information. In this reciprocal selection-updating-selection process, the lateral OFC would not function as a passive filter of sensory information, but rather as an active work pad that continuously examines the information passing through its “multimedia” recorders, by comparing it to internal, stored data and evaluating its relevance to possible actions. This more elaborate active selective function is similar in many ways to working memory. Indeed, there is some evidence that the ventrolateral PFC, including the lateral OFC, is active during specific working memory tasks in which information is associated with rewards (Kennerley and Wallis, 2009; Ronel, 2018; Wallis, 2019). Here, we suggest that the ventrolateral PFC, unlike the dorsolateral PFC, is the locus of specific reward-based working memory which serves as the foundation for its other goal-directed selection functions. Clearly, however, this hypothesis needs to be tested. Diamond’s (2005) view of ADHD without hyperactivity, and the evidence supporting her view may help ground this notion of the association between working memory functions and active selection properties of the ventrolateral PFC.

## Inattentiveness, working memory, and ventrolateral PFC

Based on her accumulating research and neurocognitive models, Diamond (2016) characterized the PFC as a key player in the exercise of executive functions. Diamond describes executive functions as the group of skills required for concentration, thinking, problem-solving, and the inhibition of automatic responses when they are evaluated negatively. Diamond argues that executive functions are similar and cooperate with, but are not identical to, self-regulation. She argues that three components constitute the core of executive functions: working memory (updating information), inhibitory control (inhibition of responses), and cognitive flexibility (shifting between responses and cognitive processes). Top-down attention, which includes selective and focused attention, is included in inhibitory control, together with the inhibition of thoughts, memories, and behavior. Working memory is defined as the function of relating a mental representation (number, fact, idea, memory, perception object, etc.) to another, thus manipulating the information to reorder, calculate and compare it. Cognitive flexibility relies on the first two components, which develop earlier in life, and is described as the ability to see something from different perspectives, switch between tasks, and switch or change a planned course of action when needed.

Diamond’s model may thus have bearing on the role of the ventrolateral PFC: is it part of inhibitory control, given its selective properties? (e.g., Ronel, 2018; Baytunca et al., 2021), or it is part of working memory, because of its mental manipulation properties? (e.g., Kennerley and Wallis, 2009; Zsuga et al., 2016).

Disentangling these two possibilities is not straightforward, since working memory and inhibition are tightly linked according to Diamond (2005, 2016) and Friedman and Miyake (2017). Working memory and selective attention are also interrelated, and it is almost impossible to differentiate between the two. The functions of working memory; namely, inhibitory control and selective attention, are hard to differentiate during childhood on both the neural and functional levels and continue to share similar neural networks and be functionally related in the adult brain (Nelson et al., 2015). Diamond (2016) reports studies showing that the ability to inhibit distractions, which is a characteristic of selective attention, has a stronger link to working memory than to inhibitory control. Thus, to date, it is difficult to determine whether selective attention is a function in its own right, or a subfunction of working memory or inhibitory control. Moreover, Postle (2006) suggests that maybe the roles are reversed, and working memory is a property of attention.

Although there is a theoretical debate regarding the dissociation and association between attention and working memory, we suggest that although it is involved in perceptual selective attention, the ventrolateral PFC does so under the umbrella of working memory. Further support for the idea that the ventrolateral PFC is engaged in both working memory and selective attention comes from research on Attention-Deficit Hyperactivity Disorder without hyperactivity (ADHD-I), which is commonly known as ADD or inattentiveness. In general, several brain regions and neural pathways were found to be involved in ADHD. Functional MRI studies have found decreased activation in the ventrolateral PFC, cerebellum, and PFC-striatal circuits, and reduced gray matter in the medial OFC (Zang et al., 2007; Cubillo et al., 2012; Norman et al., 2016; Lukito et al., 2020). Few studies, however, have attempted to distinguish between different types of ADHD on a neural basis. The studies that have done so have found a correlation between the difficulty to maintain attention, which is the core complaint of individuals with ADHD-I, and impairments in working memory (Diamond, 2005; Orinstein and Stevens, 2014; Elisa et al., 2016).

Studies comparing the neural correlates of individuals with ADHD-I and controls have failed to identify a different pattern of activity in the PFC but reported slightly higher activity in BA 10, as well as in other non-PFC areas in the brain (Orinstein and Stevens, 2014). Recall that the BA 10 corresponds to the dorsolateral PFC, which is viewed by many as the locus of general working memory processes (Kennerley and Wallis, 2009; Barbey et al., 2013; Wischnewski et al., 2021). However, the activation of a more dorsal area of the PFC could be influenced by the specific functional task or area of interest tested in these studies. For example, in Elkana et al. (in preparation, 2023), dTMS (Deep Trans Magnetic Stimulation) was centered on the dorsolateral PFC in 57 adults with ADHD. In Orinstein and Stevens’ (2014) study, the task was to identify an auditory target among distractors but did not include an update or change of this target’s value during the task. Hence, the dorsolateral PFC was active and possibly maintained the task demands active but not the neighboring ventrolateral cortex. Thus, whereas the ventrolateral PFC and the OFC are involved in selective attention and working memory, they may only do so for an input whose outcome value needs to be evaluated and updated.

## Different Types of WM and their corresponding neuroanatomical locations

Although traditionally the literature has focused on the dorsolateral PFC as the locus of working memory processes, current research on the neuroanatomical correlates of these processes has revised this view and posits that different areas mediate different working memory processes (O’Reilly and Frank, 2006; Ward, 2020; Wischnewski et al., 2021). Although the dorsolateral PFC plays a key role in a range of working memory tasks such as computation, the encoding and retrieving of verbal information, and the integration of input needed for decision-making, many other cortical and subcortical areas are considered to be involved in processes related to working memory (Chai et al., 2018). These mainly include the ACC and parietal cortex at the cortical level, and the basal ganglia and cerebellum at the subcortical level. Recent studies have pointed to the involvement of ACC in adjustments when task demands change, and the role of the basal ganglia nuclei in the focusing of attention, which appear to overlap to some extent with the ventrolateral PFC.

Hence, despite accumulating evidence, there is still no integrative model of working memory. Clearly, this type of model would shed light on the specific role of the ventrolateral PFC in updating perceptual stimuli according to the outcome value. Future research should attempt to pinpoint the specific roles of the dorsolateral PFC, the ventrolateral PFC, the ACC, and the basal ganglia.

## Discussion and conclusion

The ventrolateral PFC, and more specifically the lateral OFC, have a number of specific characteristics. The lateral OFC is known to play a role in the goal-directed selection of information. Ronel and Ben Shalom argued that this information was primarily perceptual (Ben Shalom and Bonneh, 2019). We hypothesize that the ventrolateral PFC enters into larger working memory functions and that this area may be responsible for value-based working memory. This hints that working memory may not be divided solely in terms of perceptual information (verbal as compared to spatial), as proposed by many and criticized by many others (Baddeley and Logie, 2012; Diamond, 2016; Ward, 2020). Rather, different forms of working memory with and without perceptual features may be mediated by different subareas of the lateral PFC, as well as across the brain as a whole. While most working memory research tasks correlate with the dorsolateral PFC and have established it as the locus of working memory processes, growing research evidence has revealed that other brain areas are involved with working memory such as the ACC and the cerebellum.

We believe that the ventrolateral PFC and more specifically the lateral OFC participates in, and is the locus of the outcome value of working memory.

We further believe that a new, integrative model of working memory should be explored and developed. We advocate further research focusing on the ventrolateral PFC and its functional and structural links to working memory. This model should distinguish between different working memory tasks, the brain areas involved, and the mediation of the execution of these tasks. Consistent with Diamond’s description of the difficulty differentiating between selective attention and other executive functions such as inhibition and working memory, we argue that an integrative and comprehensive neuroanatomical model of working memory should reevaluate areas and tasks that are traditionally viewed as associated with selective attention, and revisit them through the prism of goal-directed working memory processes.

The idea that the lateral OFC plays a major role in a specific working memory task calls for an update of Ben Shalom’s model of the PFC’s functional organization, and specifically the ventral PFC, which corresponds to BA 11 and 47. In line with Ronel and Ben Shalom’s hypothesis, we support the idea that this area is involved in the processing of perceptual stimuli. Nevertheless, we propose that the lateral sub-area does not merely play a role in selection and inhibition of perceptual information, but rather is involved in a more elaborate updating of information, as a function of its relevance and value to achieving an action goal. This does not require rejecting the previous model, but rather revising it. Based on Diamond (2016), we suggest that one possible reason for the confusion between selection and working memory can be attributed to their many shared functional characteristics. Another direction which should be further explored is anatomical. Whereas we focused on the lateral OFC (lateral BA 11), the ventrolateral PFC does also include BA 47. Studies stressing the role of the lateral OFC have not differentiated between these two areas, which may end up having different yet related functions. Future research should continue to examine the processes and neuroanatomical correlates of this intriguing brain area.

## Data availability statement

The original contributions presented in the study are included in the article, further inquiries can be directed to the corresponding author/s.

## Author contributions

The present manuscript was written together by OS & OE. All authors contributed to the article and approved the submitted version.

## Conflict of Interest

The authors declare that the research was conducted in the absence of any commercial or financial relationships that could be construed as a potential conflict of interest.

## Publisher’s Note

All claims expressed in this article are solely those of the authors and do not necessarily represent those of their affiliated organizations, or those of the publisher, the editors and the reviewers. Any product that may be evaluated in this article, or claim that may be made by its manufacturer, is not guaranteed or endorsed by the publisher.
